# Ultrasound assessment of the posterior circumflex humeral artery in elite volleyball players: Aneurysm prevalence, anatomy, branching pattern and vessel characteristics

**DOI:** 10.1007/s00330-016-4401-8

**Published:** 2016-06-02

**Authors:** Daan van de Pol, Mario Maas, Aart Terpstra, Marja Pannekoek-Hekman, Sena Alaeikhanehshir, P. Paul F. M. Kuijer, R. Nils Planken

**Affiliations:** 1 Department of Radiology, Academic Medical Center/University of Amsterdam, PO Box 22700, NL-1100 DE Amsterdam, The Netherlands; 2 Coronel Institute of Occupational Health, Academic Medical Center/University of Amsterdam, Amsterdam, The Netherlands

**Keywords:** Ultrasound, Posterior circumflex humeral artery, Volleyball, Aneurysm, Surveillance

## Abstract

**Objectives:**

To determine the prevalence of posterior circumflex humeral artery (PCHA) aneurysms and vessel characteristics of the PCHA and deep brachial artery (DBA) in elite volleyball players.

**Methods:**

Two-hundred and eighty players underwent standardized ultrasound assessment of the dominant arm by a vascular technologist. Assessment included determination of PCHA aneurysms (defined as segmental vessel dilatation ≥150 %), PCHA and DBA anatomy, branching pattern, vessel course and diameter.

**Results:**

The PCHA and DBA were identified in 100 % and 93 % (260/280) of cases, respectively. The prevalence of PCHA aneurysms was 4.6 % (13/280). All aneurysms were detected in proximal PCHA originating from the axillary artery (AA). The PCHA originated from the AA in 81 % of cases (228/280), and showed a curved course dorsally towards the humeral head in 93 % (211/228). The DBA originated from the AA in 73 % of cases (190/260), and showed a straight course parallel to the AA in 93 % (177/190).

**Conclusions:**

PCHA aneurysm prevalence in elite volleyball players is high and associated with a specific branching type: a PCHA that originates from the axillary artery. Radiologists should have a high index of suspicion for this vascular overuse injury. For the first time vessel characteristics and reference values are described to facilitate ultrasound assessment.

***Key Points*:**

• *Prevalence of PCHA aneurysms is 4.6 % among elite volleyball players.*

• *All aneurysms are in proximal PCHA that originates directly from AA.*

• *Vessel characteristics and reference values are described to facilitate US assessment.*

• *Mean PCHA and DBA diameters can be used as reference values.*

• *Radiologists need a high index of suspicion for this vascular overuse injury.*

## Introduction

Elite overhead athletes, like volleyball players, are at risk of ischaemic digits due to arterial emboli originating from an aneurysmal and thrombosed proximal posterior circumflex humeral artery (PCHA) in the dominant shoulder, although the exact prevalence among these athletes is unknown [1]. Although ultrasound (US) is the first-line imaging modality for assessment of the PCHA, identifying and assessing the PCHA is a cumbersome process in the hands of radiologists and vascular technologists.

**Fig. 1:**
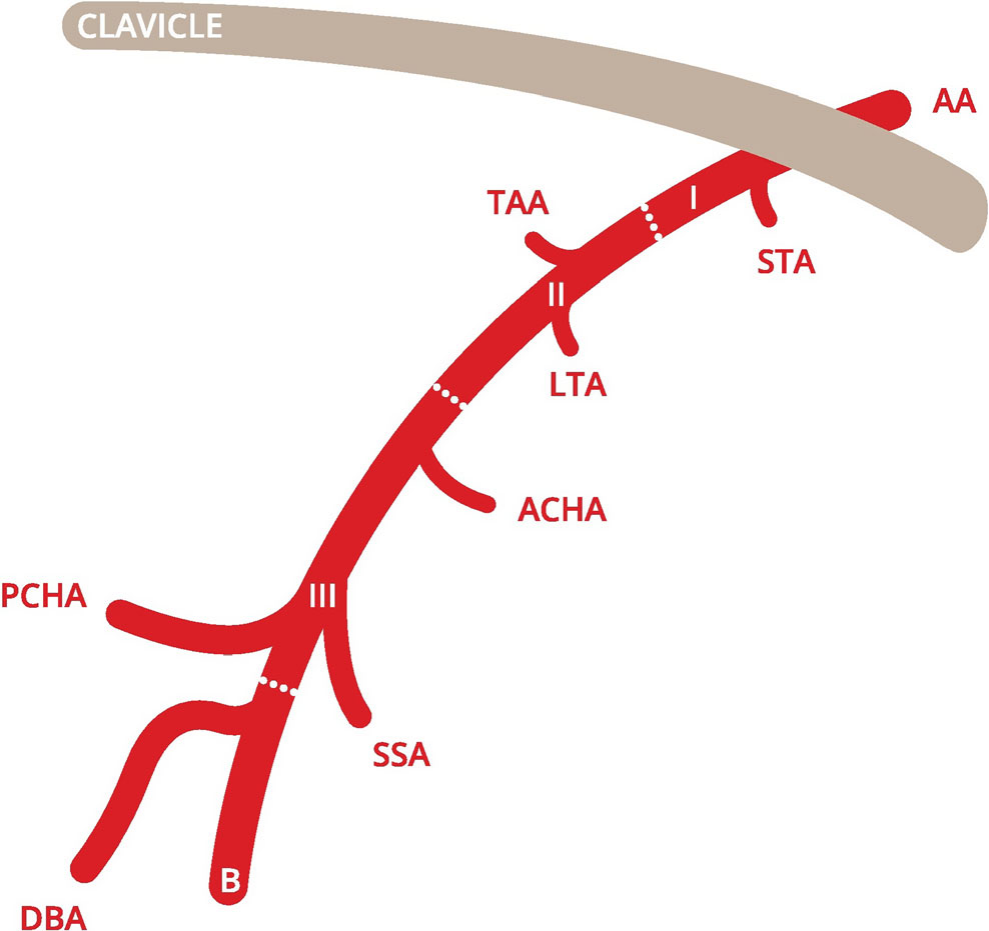
Classic PCHA origin from the axillary artery [7]. *AA* axillary artery, *I* first part of axillary artery, *II* second part of axillary artery, *III* third part of axillary artery, *B* brachial artery, *STA* superior thoracic artery, *TAA* thoracoacromial artery, *LTA* lateral thoracic artery, *ACHA* anterior circumflex humeral artery, *SSA* subscapular artery, *PCHA* posterior circumflex humeral artery, *DBA* deep brachial artery. This figure is drafted by Mr. K.F. de Geus

**Fig. 2:**
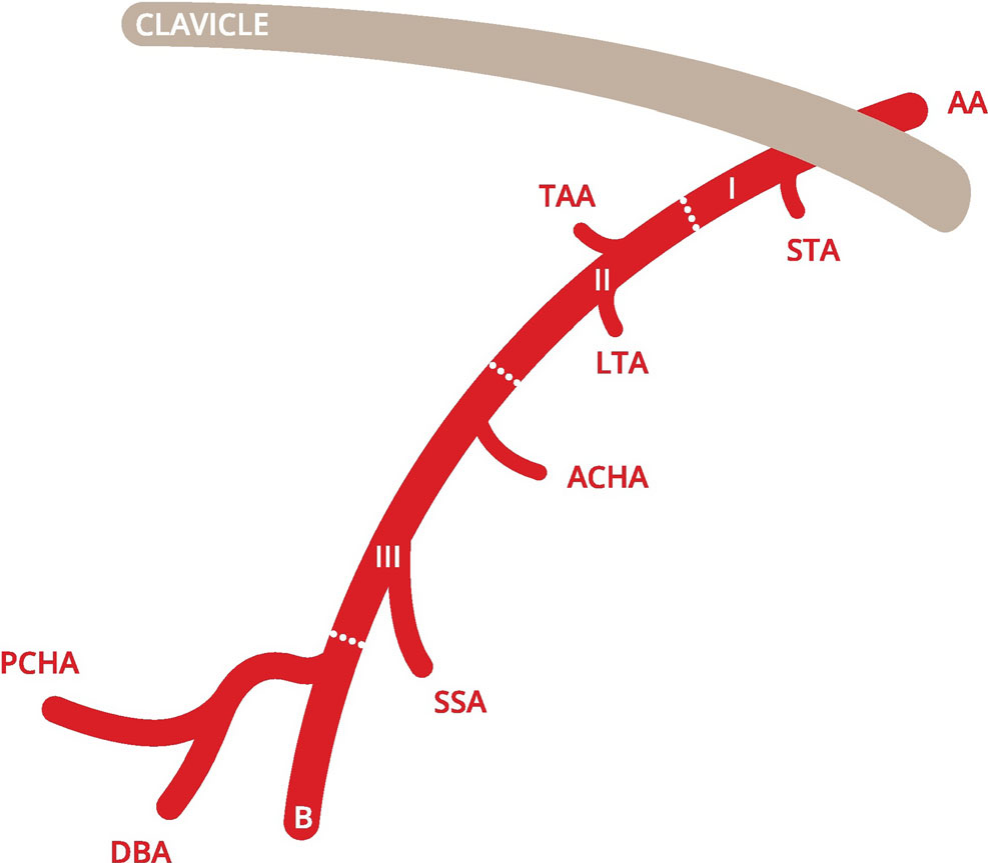
Common trunk of the PCHA and DBA [7]. *AA* axillary artery, *I* first part of axillary artery, *II* second part of axillary artery, *III* third part of axillary artery, *B* brachial artery, *STA* superior thoracic artery, *TAA* thoracoacromial artery, *LTA* lateral thoracic artery, *ACHA* anterior circumflex humeral artery, *SSA* subscapular artery, *PCHA* posterior circumflex humeral artery, *DBA* deep brachial artery. This figure is drafted by Mr. K.F. de Geus

The PCHA is a relatively small branch originating from the third part of the axillary artery (AA). Although it is frequently the last branch originating from the AA, with a prevalence of origin variations reported to be 33 − 42 % [2, 3], the deep brachial artery (DBA), which normally originates from the proximal brachial artery, may have an aberrant origin and also arise from the dorsal AA, near to and closely resembling the PCHA (Figs. 1 and 2) [4–6]. Since the PCHA is prone to injury in overhead athletes where the DBA has not been reported to be at risk in overhead athletes in the medical literature, it is important to distinguish between the PCHA and the DBA [7].

The SPI-US protocol (Shoulder PCHA pathology and digital Ischemia – UltraSound protocol) can be used to assess PCHA and DBA anatomy, branching pattern, diameter measurement and detection of aneurysms [7, 8]. However, reference values for arterial diameters should be considered when reporting aneurysms in accordance with the suggested standards for reporting on aneurysms by Johnson et al. [9]. The data regarding normal PCHA diameters are not yet published in the medical literature.

Data on normal and aneurysmal PCHA diameters and arterial characteristics would facilitate the accurate identification and assessment of the PCHA, and could be used as reference values for aneurysmal and normal vessels in clinical assessment and for research purposes. For other peripheral aneurysms, such as the common femoral artery and the popliteal artery, similar data is commonly used for diagnostic and therapeutic purposes [10, 11].

The purpose of this study, therefore, is (1) to determine the prevalence of PCHA aneurysms in elite volleyball players, and (2) to describe PCHA and DBA characteristics that can be used to accurately identify and assess the PCHA.

## Materials and methods

### Study design

A cross-sectional ultrasound study was performed among elite volleyball players active at national and international top level from January to July 2014. Official approval was granted by the Institutional Review Board (IRB) at our academic hospital and permission was obtained from the Institutional Review Board at the Fédération Internationale de Volleyball (FIVB).

### Participants

Participants were recruited in cooperation with the FIVB and the Dutch Volleyball Association (Nevobo). Those eligible for inclusion were all elite male indoor volleyball players active in the Dutch national top league, second league or Dutch national volleyball team in the season 2013 − 2014, and all elite male and female beach volleyball players active during the main tournament of the 2014 Beach Volleyball Grand Slam Tournament in The Hague. Volleyball players were not considered eligible for inclusion in the case of a positive history for vascular surgery of the dominant shoulder, use of cardiovascular medication or lack of written informed consent.

### Ultrasound assessment

All US examinations were performed by one of two registered vascular technologists (RVTs) using a Dynamic LOGIQ-e (General Electric Company 2006) scanner equipped with a 12 L-RS linear array transducer probe (5-13 MHz), following the standardized SPI-US protocol [8], which enables accurate and sonographer-independent PCHA and DBA diameter measurements with excellent inter-observer agreement [7]. Both RVTs had more than 20 years experience with vascular US, had studied the anatomy of the branching pattern of the AA and its anatomical variations intensively, and were experienced in conducting the US protocol that was used.

US examination included assessment of branching pattern (origin variations), local anatomy (artery course at the origin) and determination of proximal PCHA and DBA diameters (measured at 1 centimetre (cm) distance from the origin). Arterial diameters were measured on cross-sectional greyscale B-mode images. In addition, participants were screened for the presence of PCHA aneurysms. Aneurysms were defined as a segmental vessel diameter increase ≥1.5, and segmental vessel diameter increase between ≥1 and ≤1.5 was defined as dilatation [9], In the event of intravascular thrombus or stenosis, colour Doppler was used to confirm the presence of thrombus by no flow regions, and waveform characteristics were obtained to visualize a triphasic or blunted signal.

In a later phase, the obtained US data were independently reviewed by both RVTs and classified as normal, doubtful or pathological. In the case of divergent conclusions, both RVTs discussed these data in order to reach consensus. Finally, US images of all pathological and doubtful cases were reviewed, discussed and definitely classified as normal or pathological during consensus meetings in which both RVTs and a vascular radiologist participated.

### Data analysis

Data were entered in SPSS (version 21.0, 2012, SPSS Inc.). A p-value ≤0.05 was considered significant in all tests. The mean, standard deviation, minimum and maximum of age, body height and body surface area (BSA) were reported for men and women separately. Body surface area was calculated according to Du Bois’ formula (BSA cm^2^ = weight^0.425^ kg * height^0.725^ cm * 71.84) [12].

The proximal course (defined as parallel or curved), and the prevalence and type of PCHA and DBA origin variations were reported for the group as a whole.

The mean, standard deviation, minimum and maximum of normal and aneurysmal PCHA and DBA diameters were reported in millimetres (mm) and corrected for BSA in mm per square metre (m^2^). The intra-participant PCHA-DBA diameter ratio was calculated to objectify the interdependence, and the intra-participant PCHA diameter ratio was calculated to objectify increase in segmental vessel diameter.

## Results

### Participants

From January to July 2014, a total of 281 elite volleyball players were assessed using the standardised SPI-US protocol. One player was excluded from the study due to a history of PCHA surgery in the dominant shoulder. As a result, 280 elite volleyball players were included: 245 men and 35 women. Male participants were on average 25 ± 5 years old (range: 17 − 41 years), had a body height of 194 ± 7 cm (range: 170 − 212 cm), and a BSA of 2.16 ± 0.1 m^2^ (range: 1.77 − 2.61 m^2^). Female participants were on average 26 ± 4 years old (range: 17 − 32 years), had a body height of 180 ± 6 cm (range: 169 − 196 cm) and a BSA of 1.87 ± 0.1 m^2^ (range: 1.71 − 2.09 m^2^).

### Aneurysm prevalence and diameters of aneurysmal and normal PCHA and DBA

**Fig. 3:**
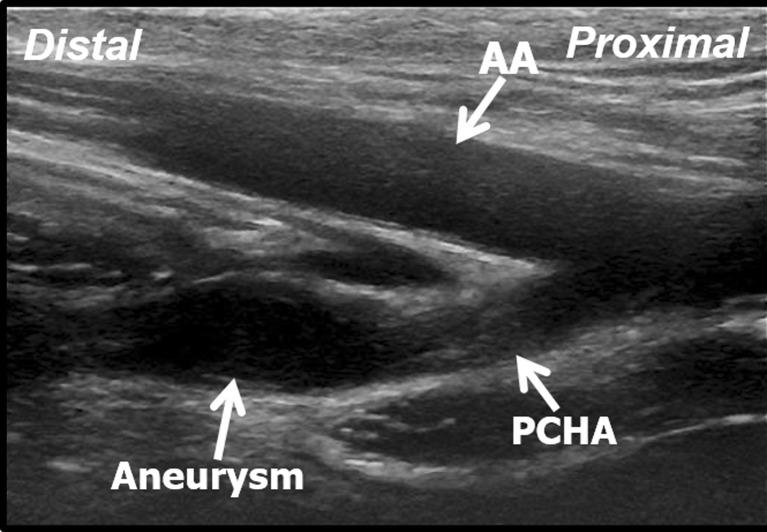
Longitudinal B-mode ultrasound image of the aneurysmatic proximal PCHA in a 31-year-old professional volleyball player. *AA* axillary artery, *PCHA* posterior circumflex humeral artery

**Fig. 4:**
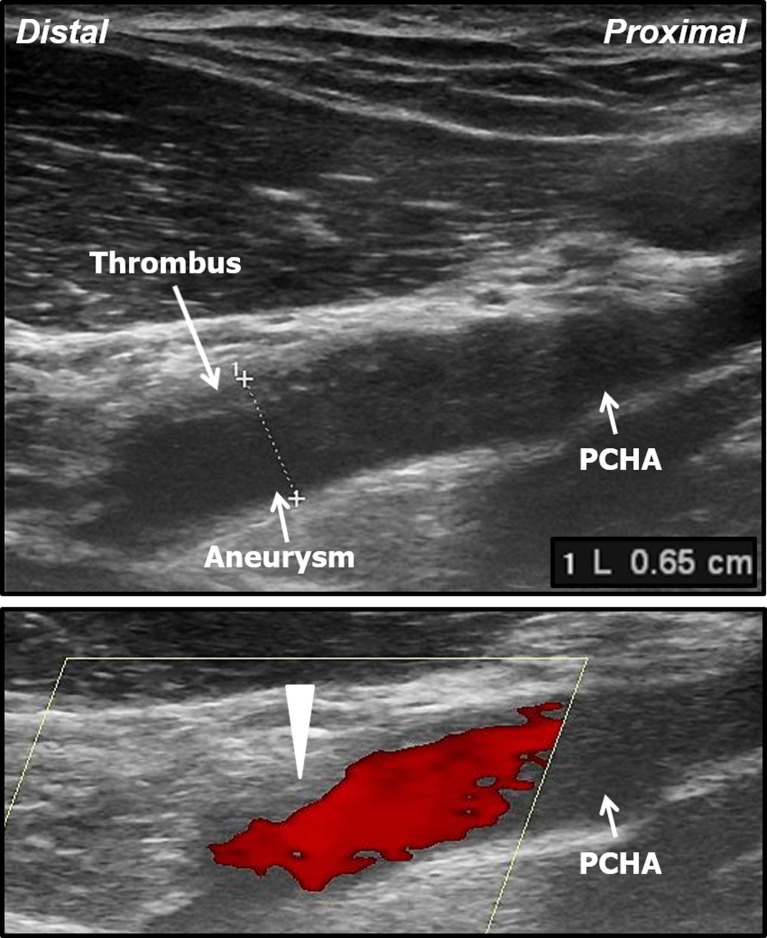
Upper panel: longitudinal B-mode ultrasound image of the aneurysmatic proximal PCHA with intravascular thrombus in a 29-year-old professional volleyball player. **Lower panel:** colour Doppler ultrasound image of the aneurysmatic proximal PCHA, note there is no colour flow in the thrombus region (arrowhead). *PCHA* posterior circumflex humeral artery

In total, 17 PCHA abnormalities were detected – 13 aneurysms (11 in men and two in women), three dilatations (all in men) and one occlusion (in a man) (4.6 % (13/280) prevalence of PCHA aneurysms). All participants were informed about the outcome of the US assessment and participants with pathological US findings were advised about follow-up. All aneurysms were detected in a PCHA that originated directly from the axillary artery. Ten aneurysms (77 %) were found in the most proximal PCHA vessel segment within 1 cm from the origin out of the axillary artery (Fig. 3). All aneurysms were fusiform-shaped and showed arterial wall irregularities. In four cases the PCHA demonstrated a tortuous course. Intravascular thrombus was visualised in three cases (Fig. 4). Characteristics of the PCHA aneurysm are listed per volleyball player in Table 1.

**Table 1:** Characteristics of the posterior circumflex humeral artery (PCHA) aneurysm per volleyball player

Sex	Age (years)	Body surface area (m^2^)	Aneurysmal PCHA diameter (mm)	Aneurysmal PCHA diameter corrected for body surface area (mm/m^2^)	Normal PCHA diameter (mm)	Normal PCHA diameter corrected for body surface area (mm/m^2^)	Intra-participant PCHA diameter ratio	Aneurysm − distance to origin (mm)	Aneurysm − shape	Aneurysm − arterial wall complications	Aneurysm – presence of intravascular thrombus
M	32	2.11	9.9	4.69	4.3	2.04	2.30	4	Tortuous, fusiform	Irregular	No
M	34	2.22	7.8	3.51	3.4	1.53	2.29	8	Fusiform	Irregular, thickened	Yes
M	28	2.27	5.5	2.42	2.9	1.28	1.90	15	Fusiform	Irregular	No
M	19	1.99	4.3	2.17	2.3	1.16	1.87	6	Tortuous, fusiform	Irregular	No
M	29	1.83	4.2	2.29	2.3	1.26	1.83	5	Fusiform	Irregular	No
M	29	2.17	6.5	3.00	3.5	1.61	1.86	8	Fusiform	Irregular, thickened	Yes
M	32	2.14	5.9	2.75	3.4	1.59	1.74	6	Tortuous, fusiform	Irregular, thickened	Yes
M	25	2.07	6.1	2.95	3.6	1.74	1.69	8	Fusiform	Irregular, thickened	No
M	23	2.25	4.7	2.09	3.0	1.33	1.57	5	Fusiform	Irregular	No
F	29	1.78	5.0	2.80	3.2	1.79	1.56	12	Fusiform	Irregular	No
M	27	2.17	5.9	2.72	3.8	1.75	1.55	7	Tortuous, fusiform	Irregular	No
F	30	1.79	5.3	2.96	3.5	1.96	1.51	8	Fusiform	Irregular	No
M	31	2.13	4.8	2.25	3.2	1.50	1.50	13	Fusiform	Irregular	No

**Fig. 5:**
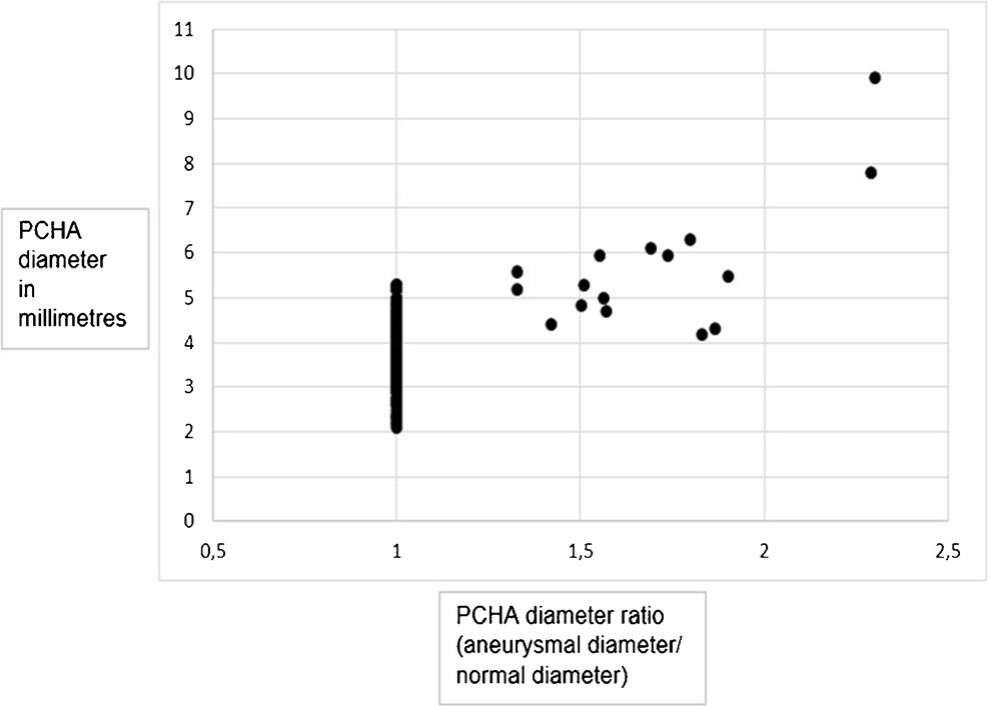
Scatter plot of absolute posterior circumflex humeral artery (PCHA) diameters in millimetres

**Fig. 6:**
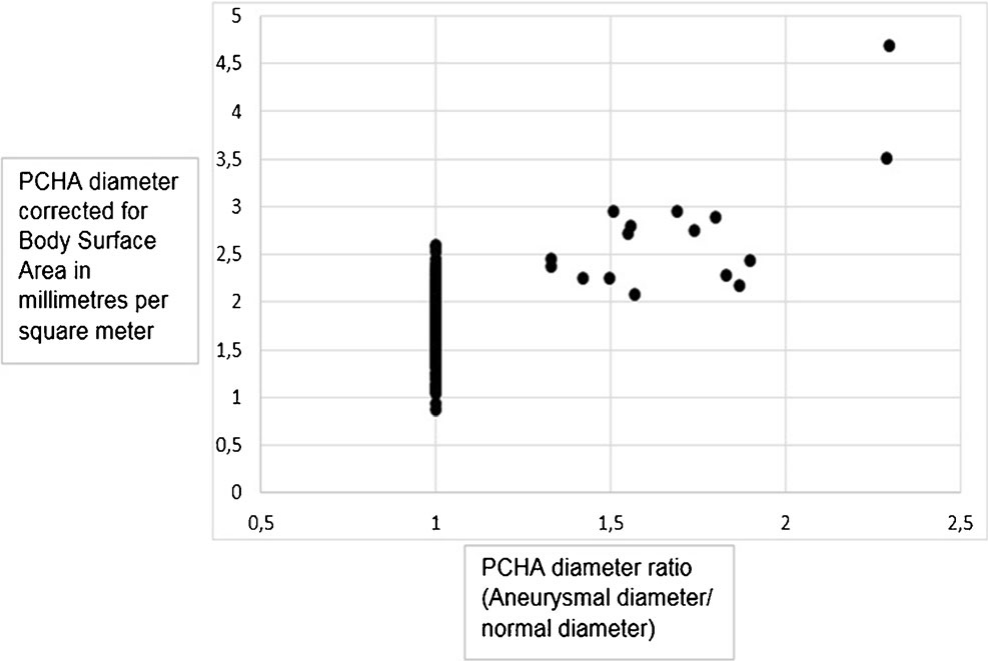
Scatter plot of posterior circumflex humeral artery (PCHA) diameters corrected for body surface area in millimetres per square metre

The mean aneurysm PCHA diameter in men was 5.9 mm ± 1.7 (95 % CI 4.8 − 7.1), and 5.2 mm ± 0.2 (95 % CI 3.2-7.1) in women. Corrected for BSA, the diameter for men and women was 2.8 mm/m^2^ ± 0.8 and 2.9 mm/m^2^ ± 0.1, respectively (Table 2). These diameters were significantly greater compared to non-dilated PCHA vessel segments (p < 0.01) (Figs. 5 and 6).

**Table 2:** Normal and aneurysmal PCHA and DBA diameters

	Normal diameter				Aneurysmal diameter			
	Men		Women		Men		Women	
	n	mean	n	mean	n	mean	n	mean
PCHA								
in millimetres	245	3.8 ± 0.6	35	3.5 ± 0.6	11	5.9 ± 1.7	2	5.2 ± 0.2
corrected for body surface area in millimetres per square metre		1.8 ± 0.3		1.8 ± 0.4		2.8 ± 0.8		2.9 ± 0.1
DBA								
in millimetres	225	2.3 ± 0.5	35	2.0 ± 0.5	n.a.		n.a.	
corrected for body surface area in millimetres per square metre		1.1 ± 0.2		1.1 ± 0.2				

*n.a.* not applicable

All DBAs showed a smooth calibre over the proximal course without any dilatations or aneurysmal segments.

In total, the diameters of 280 normal PCHA vessel segments were measured, with a mean diameter of 3.8 mm ± 0.6 (95 % CI 3.7 − 3.9) in 245 men, and 3.5 mm ± 0.6 (95 % CI 3.3 − 3.7) in 35 women. Corrected for BSA, the diameter for men and women was 1.8 mm/m^2^ ± 0.3 and 1.8 mm/m^2^ ± 0.4, respectively (Table 2).

The diameters of 260 normal DBA vessel segments were measured with a mean diameter of 2.3 mm ±0.5 (95 % CI 2.2 − 2.3) in 225 men, and 2.0 mm ± 0.5 (95 % CI 1.9 − 2.2) in 35 women. Corrected for BSA, the diameter for both men and women was 1.1 ± 0.2 (Table 2). The intra-participant PCHA-DBA diameter ratio was >1 in all these participants. Diameters of 20 DBAs were unable to be determined due to absence in the axillary pit or as a result of origin variation leading to insufficient imaging quality.

### Anatomy, branching pattern and course of PCHA and DBA

The PCHA was identified in 100 % of cases and the DBA in 93 % (n = 260). For the 7 % of cases (n = 20) in which the DBA was not identified in the axillary pit, the cause might be due to a more distal origin from the brachial artery, an anatomical variant with an absent DBA, or because it was being overlooked by the vascular technologist.

An anatomical variation of the PCHA was found in 52 of 280 cases (19 %), and included a common trunk with the DBA (n = 16), a common trunk with a different artery from the DBA (n = 18), a common trunk with two other arteries (n = 10), and a trunk with a proximal origin that was not visualised (n = 8). The DBA was found to arise from a common trunk in 70 of 260 cases (27 %).

**Fig. 7:**
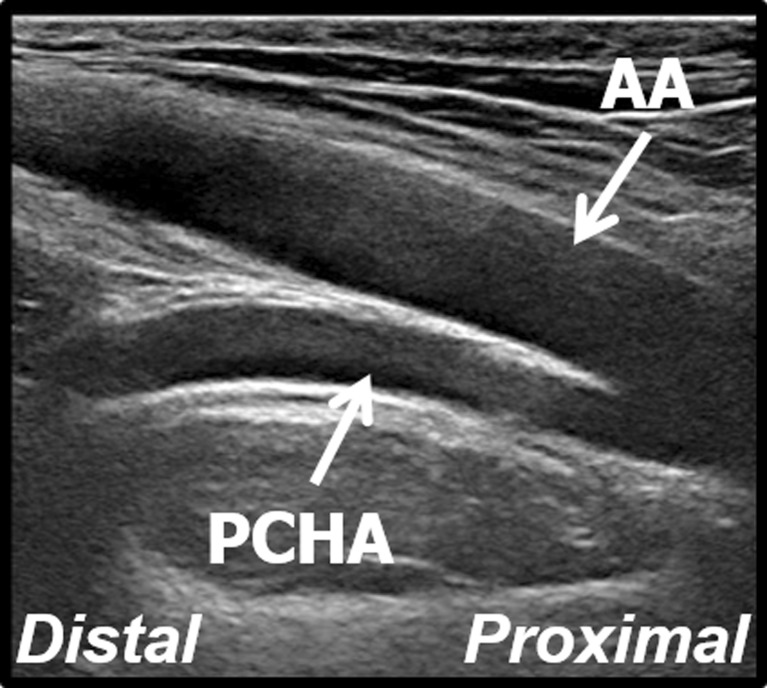
Longitudinal B-mode ultrasound image of the view of the PCHA with a curved course dorsally towards the humeral head. *AA* axillary artery, *PCHA* posterior circumflex humeral artery

**Fig. 8:**
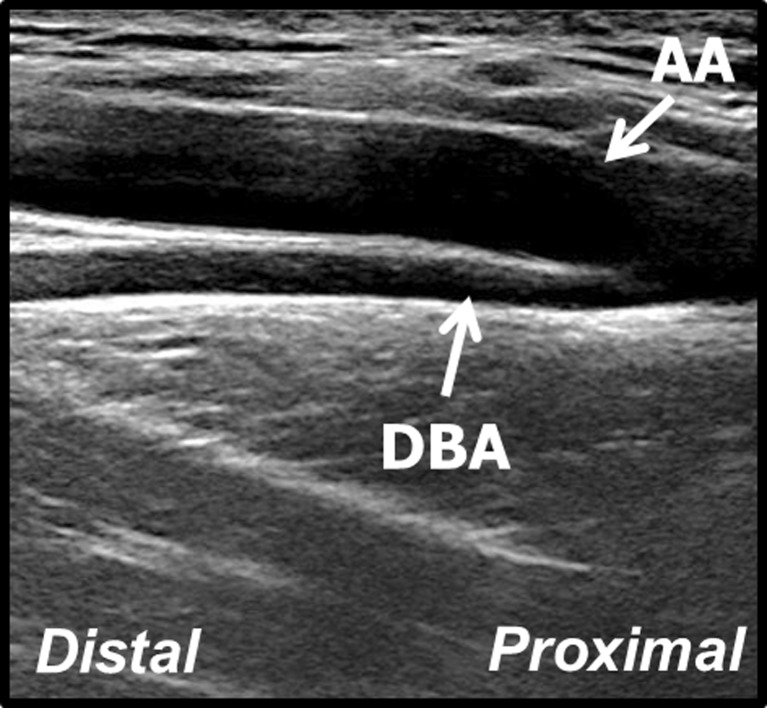
Longitudinal B-mode ultrasound image of the view of the DBA with a straight course parallel to the axillary artery. *AA* axillary artery*, DBA* deep brachial artery

The PCHA showed a curved course dorsally towards the humeral head in 93 % of the normal anatomical variants (211/228) (Fig. 7), and 7 % could not be determined due to insufficient imaging (n = 17). The DBA showed a straight course parallel to the axillary artery in 93 % of the normal anatomical variants (177/190) (Fig. 8), and 7 % could not be determined due to insufficient imaging (n = 13). The proximity of the PCHA origin seemed to determine the degree and level of curvature. A proximal PCHA origin from the axillary artery led to a more distal curve towards the humeral head (e.g. after 2 to 3 cm), whereas a more distal origin led to an instant and more sharp curve. The DBA course did not seem to be influenced by the proximity of the origin. No association was found between the degree and level of curvature of the PCHA and aneurysm prevalence.

In the case of an origin variation, the PCHA and DBA course proved more difficult to determine. In 52 PCHA origin variants, 73 % showed a curved course towards the humeral head (n = 38), and 27 % could not be determined due to insufficient imaging (n = 14). In 70 DBA origin variants, 69 % showed a straight course parallel to the axillary artery (n = 48), and 31 % could not be determined due to insufficient imaging (n = 22). An overview of PCHA and DBA characteristics is shown in Tables 3.

**Table 3:** Overview of posterior circumflex humeral artery (PCHA) and deep brachial artery (DBA) vessel characteristics and diameters

	PCHA	DBA
Origin	Dorsal of the axillary artery, proximal of the DBA	Dorsal of the axillary artery, distal of the PCHA
Course	Curved towards the dorsal side of the humerus	Straight and parallel to the axillary artery
Presence in axillary pit	Always present	Commonly present (absent in 7 % of cases)
Intra-individual ratio	>1.0 (PCHA dm/DBA dm)	<1.0 (DBA dm/PCHA dm)
Average diameter (in millimetres)	3.8 (men) 3.5 (women)	2.3 (men) 2.0 (women)
Average diameter (corrected for Body Surface Area in millimetres per square meter)	1.8 (men) 1.8 (women)	1.1 (men) 1.1 (women)
Originating directly from the axillary artery	81 % of cases	75 % of cases

*dm* diameter

## Discussion

The prevalence of proximal PCHA aneurysms in elite volleyball players is high and associated with a specific branching type, namely a PCHA that originates directly from the axillary artery. In contrast, no PCHA aneurysms were detected in anatomical variants such as a common trunk of the PCHA and DBA. The DBA was normal in all athletes and no DBA aneurysms were detected. The described vessel characteristics enable a distinction to be made between the PCHA and DBA, where the PCHA is larger and has a curved course dorsally towards the humeral head.

The PCHA was present in the axillary pit in 100 % of cases, arose from a common trunk in 19 %, and showed a curved course dorsally towards the humeral head in 93 % of cases. The average normal PCHA diameter was 3.8 mm for men, 3.5 mm for women and, corrected for BSA, 1.8 mm/m^2^ for both. For the aneurysmal PCHA, these diameters were 5.9 mm for men and 5.1 for women. All aneurysms were detected in a PCHA that originated directly from the axillary artery. All DBAs showed a smooth calibre over the proximal course without any aneurysmal segments. The DBA was present in the axillary pit in 93 % of cases, arose from a common trunk in 25 %, and showed a straight course parallel to the axillary artery in 93 % of cases. The average normal DBA diameter was 2.3 for men, 2.0 for women and 1.1 mm/m^2^ for both. The intra-individual PCHA-DBA diameter ratio was ≥1.0 in all participants.

Worldwide elite overhead athletes, like volleyball players, are potentially at risk of ischaemic digits due to arterial emboli originating from an aneurysmal and thrombosed PCHA in the dominant shoulder. The incidence of PCHA aneurysms in this specific population is high, as shown by the current study. Several studies reported about PCHA pathology with serious ischaemic complications among elite volleyball players [1, 13–24]. Also, multiple cases have been reported in elite baseball pitchers [13, 25–31], tennis players [17, 18, 32], swimmers[33], kayakers[34], yoga practitioners [35], trapeze flying artists [20], American football players [32] and even one in regular work, namely a mechanic [33]. No PCHA pathology was detected in some 350 examined PCHAs of healthy subjects [3, 36, 37]. Several studies suggest that repetitive powerful overhead movements in volleyball, like spiking and serving, cause chronic vessel wall injury as a result of positional traction and compression of the proximal PCHA [14, 15, 21]. This cumulative PCHA trauma can cause a continuum of pathology ranging from local intimal hyperplasia to vessel widening of <150 % (dilatation) and >150 % (aneurysm), and occlusion [30].

Identification of PCHA aneurysms at an early stage might prevent thromboembolic complications and irreversible tissue damage [1]. Potential therapeutic options include surgical ligation and endovascular coiling [14], while conservative treatment consists of cessation of sports activities [38]. However, identification and assessment of the PCHA is cumbersome in the hands of radiologists and vascular technologists due to anatomical variations and the very similar DBA originating nearby. The reported PCHA and DBA vessel characteristics enable easy and reliable PCHA and DBA identification and discrimination using US. This information facilitates accurate US assessment of the PCHA and DBA in both a clinical and screening setting. We expect the accuracy of the SPI-US protocol to improve when the diameters and arterial course are considered by radiologists and vascular technologists. Furthermore, we provide reference values for normal and aneurysmal PCHA diameters for male and female elite volleyball players. When corrected for BSA, the values for male and female volleyball players are comparable.

### Normal and aneurysmal PCHA and DBA diameters

Normal values for arteries prone to aneurysm formation are commonly determined using US, and are currently used for diagnostic and therapeutic purposes [11, 39]. Diameters of normal PCHA vessel segments were homogenous with a small standard deviation. However, the normal PCHA vessel segment diameter differed for males and females. When corrected for BSA, these diameters were comparable for male and female volleyball players. This implies that unisex PCHA reference values can be used when absolute diameters are corrected for BSA.

### Aneurysm characteristics

**Fig. 9:**
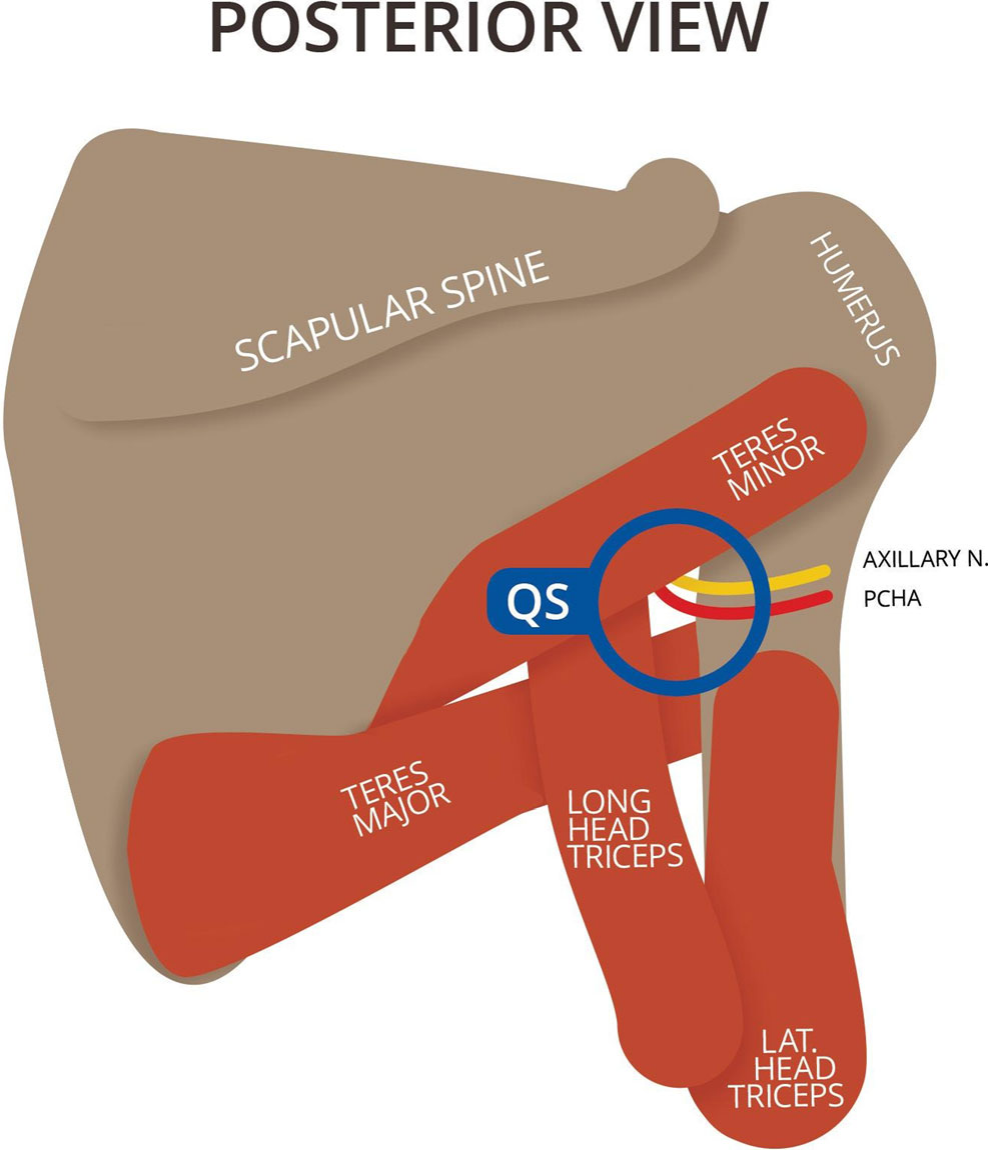
Diagrammatic representation of the quadrilateral space from posterior [7]. *QS* quadrilateral space. This figure is drafted by Mr. K.F. de Geus

Aneurysm characteristics such as the site (anatomical segment), morphological features (e.g. shape and arterial wall complications) and clinicopathological manifestation (e.g. thrombotic occlusion and embolisation) should be reported according to the suggested standards for reporting on aneurysms by Johnson et al. [9]. Classification by anatomical segment is important since aneurysms located in different sites may be associated with variations in their natural history [9]. Ten aneurysms originated within 8 mm from the origin, while the remaining three were detected between 12 and 15 mm of the origin. We hypothesized these aneurysms to have a similar aetiology, since the slightly more distal site of injury is presumably due to a more proximal PCHA origin in the axillary pit. A different aetiology, for instance entrapment of the vessel in the quadrilateral space (Fig. 9), is unlikely since the midsection of the PCHA is traversing the quadrilateral space, while the lesions were seen in the proximal part of the vessel. In this case, the axillary nerve accompanies the PCHA in the neurovascular bundle several cm distal to the origin and was not visualised [21]. Morphological features of all 13 aneurysms comprised vessel wall irregularities and a fusiform shape. Also, four aneurysmal PCHAs showed a tortuous course, which might be correlated to increased symptoms as is seen in aneurysms of the popliteal artery [40]. Lastly, intravascular thrombus was detected in three aneurysms, a source of distal embolisation, and thrombotic digital occlusion [1, 9]. Larger aneurysms are more likely to contain thrombus [40], although this was not supported by our results. However, it is possible that intravascular thrombus was not visualised due to recent embolisation during overhead movements [14, 21], possibly resulting in false negatives during the US examination.

### Anatomy, branching pattern and course of PCHA and DBA

Thorough knowledge of the possible anatomical variations of the axillary artery and its tributaries is vital when assessing the PCHA and DBA. The prevalence of PCHA origin variations is up to 33 − 42 % in the medical literature [2, 3], and 19 % in our study. Interestingly, in the current study all thirteen aneurysms were detected in PCHAs that originated directly from the axillary artery. This is consistent with the location of pathology seen in volleyball players who were diagnosed and treated in our academic hospital [1, 14, 20, 21], as well as in other reports on PCHA aneurysms in the medical literature [13, 15, 19, 22, 23, 34, 41]. This implies that a PCHA originating directly from the axillary artery is a risk factor for the development of PCHA aneurysms and thrombosis and that variant anatomy might be protective against aneurysm and thrombus formation. Among almost 350 examined PCHAs of healthy subjects no PCHA pathology was detected [3, 36, 37]. Since the PCHA curves towards the humeral head and the DBA proceeds straight and parallel to the axillary artery in most cases of both the normal branching types and anatomical variants, it is vital to objectify the PCHA curve in both the longitudinal and the transversal plane for positive PCHA identification [8].

### Strengths, weaknesses and future studies

A strength of the current study is that normal PCHA diameters were determined in a large group of elite volleyball players, the population at risk [9]. Another strength is the thorough process of data reviewing by multiple experts, since this process had contributed to an optimal classification of the collected data. A weakness of the current study is that in 7 % of cases (n = 20) the DBA was not identified in the axillary pit, which might be due to a more distal origin from the brachial artery, an anatomical variant with an absent DBA, or because it was overlooked by the vascular technologists. Therefore, the prevalence of DBA pathology remains uncertain in these athletes, although DBA abnormalities do not seem likely considering the available data in the current study.

Future studies need to assess the clinical value of PCHA screening by US in this specific population to determine the relation between symptoms and PCHA aneurysms as detected by US, since most peripheral aneurysms are known to be asymptomatic [10].

In conclusion, the prevalence of PCHA aneurysms in elite volleyball players is high and associated with a specific branching type, namely a PCHA that originates directly from the axillary artery. Radiologists should have high index of suspicion for this vascular overuse injury among elite volleyball players. The described PCHA and DBA vessel characteristics provide clear guidance to identify and assess the PCHA and DBA. Only the PCHA needs to be screened for aneurysms, which can be easily detected using ultrasound. The high prevalence of detected PCHA aneurysms asks for active policy on prevention and periodic surveillance that is easily performed using the provided PCHA and DBA characteristics.

This study is the first to provide radiologists and vascular technologists with insight knowledge into how ultrasound can be used for surveillance of a vascular overuse injury which is highly prevalent in elite overhead athletes, like volleyball players.

## Abbreviations

AA: Axillary artery
BSA: Body surface area
cm: Centimetres
DBA: Deep brachial artery
FIVB: Fédération Internationale de Volleyball
m^2^: Square metre
mm: Millimetres
mm/m^2^: Millimetres per square metre
Nevobo: Dutch Volleyball Association
PCHA: Posterior circumflex humeral artery
RVT: Registered vascular technicians
SPI-US Protocol: Shoulder vascular Pathology with digital Ischemia–UltraSound protocol
US: Ultrasound
